# Modifiable exposures to air pollutants related to asthma phenotypes in the first year of life in children of the EDEN mother-child cohort study

**DOI:** 10.1186/1471-2458-13-506

**Published:** 2013-05-24

**Authors:** Cailiang Zhou, Nour Baïz, Tuohong Zhang, Soutrik Banerjee, Isabella Annesi-Maesano

**Affiliations:** 1 Université Pierre et Marie Curie, Paris 6, Sorbonne Universités, UMR S 707: EPAR (Epidémiologie des maladies allergiques et respiratoires), Medical School Saint-Antoine, Paris, France; 2 INSERM U 707: EPAR, Paris, France; 3 School of Public Health, Peking University, Beijing, China

**Keywords:** Environment, Traffic-related air pollution, Environmental Tobacco Smoke (ETS), Pets, Moulds, Asthma, Children

## Abstract

**Background:**

Studies have shown diverse strength of evidence for the associations between air pollutants and childhood asthma, but these associations have scarcely been documented in the early life. The purpose of this study was to evaluate the impacts of various air pollutants on the development of asthma phenotypes in the first year of life.

**Methods:**

Adjusted odds ratios were estimated to assess the relationships between exposures to air pollutants and single and multi-dimensional asthma phenotypes in the first year of life in children of the EDEN mother-child cohort study (n = 1,765 mother-child pairs). The Generalized Estimating Equation (GEE) model was used to determine the associations between prenatal maternal smoking and *in utero* exposure to traffic-related air pollution and asthma phenotypes (data were collected when children were at birth, and at 4, 8 and 12 months of age). Adjusted Population Attributable Risk (aPAR) was estimated to measure the impacts of air pollutants on health outcomes.

**Results:**

In the first year of life, both single and multi-dimensional asthma phenotypes were positively related to heavy parental smoking, traffic-related air pollution and dampness, but negatively associated with contact with cats and domestic wood heating. Adjusted odds ratios (aORs) for traffic-related air pollution were the highest [1.71 (95% Confidence Interval (CI): 1.08-2.72) for ever doctor-diagnosed asthma, 1.44 (95% CI: 1.05-1.99) for bronchiolitis with wheezing, 2.01 (95% CI: 1.23-3.30) for doctor-diagnosed asthma with a history of bronchiolitis]. The aPARs based on these aORs were 13.52%, 9.39%, and 17.78%, respectively. Results persisted for prenatal maternal smoking and *in utero* exposure to traffic-related air pollution, although statistically significant associations were observed only with the asthma phenotype of ever bronchiolitis.

**Conclusions:**

After adjusting for potential confounders, traffic-related air pollution *in utero* life and in the first year of life, had a greater impact on the development of asthma phenotypes compared to other factors.

## Background

The prevalence of asthma has increased constantly in recent decades [1-3] and different factors have been implicated in its aetiology. Although genetic factors are important determinants of asthma [4], exposures to certain environmental factors play an essential role in the phenotypic expression of this condition [5,6] thus contributing substantially to the risk of its development [7].

The strength of evidence for the associations between exposures to various air pollutants and the development of childhood asthma is diverse [8]. Robust data casually related asthma to allergens including aero-allergens such as house dust mite [9]. Studies consistently demonstrated that Environmental Tobacco Smoke (ETS) increases the risk of asthma in children [10-12]. Other investigations showed an independent effect of dampness and moulds on the development of childhood asthma [13,14], although some investigations argued that the relationship may be partly explained by reporting bias [15]. Reversely, a protective effect was suggested for early life exposure to pets [16,17], exposure to domestic wood heating and rural living [18]. In contrast with initial findings [19], recent investigations, including birth cohort studies, have indicated a link between exposure to outdoor air pollution and subsequent asthma development [20-22]. Indoor air pollutants have also been related to asthma [23], but there is a lack of longitudinal data in this respect.

The aim of our study is to investigate the impacts of exposures to passive smoking, traffic-related air pollution, dampness, visible moulds, bleaching agents, house dust mites, pets and domestic heating on the development of asthma in the first year of life, which is a period of life when the lung is still developing. For passive smoking and traffic-related air pollution, *in utero* exposures were taken into account to better understand the development of childhood asthma. The health outcomes we considered in this study included both single and multi-dimensional asthma phenotypes at 0–4 months, 4–8 months, and 8–12 months respectively as well as their evolution during the first year of life. To this extent, we used data from a birth cohort to study the impacts of the *in utero* and first year of life exposures on asthma phenotypes in the first year of life.

## Methods

### Study population

The EDEN mother-child cohort study (Study of pre- and post-natal determinants of children’s growth and development) is an on-going investigation in two study centres, Nancy and Poitiers, in France. This study enrolled 2,002 women during pregnancy and was described in more detail previously [24]. This study was approved by the ethical committee (*Comité Consultatif pour la Protection des Personnes dans la Recherche Biomédicale*, CCPPRB) of Kremlin Bicêtre and by the Data Protection Authority (*Commission Nationale de l’Informatique et des Libertés*, CNIL). Two hundred and thirty-seven children failed to be followed-up at 1 year of age.

### Data collection

Data were collected when children were at birth, and at 4, 8 and 12 months of age respectively, by standardised questionnaires that allowed the investigation of socio-demographic characteristics, *in utero* and first year of life environmental exposures and respiratory health of the children throughout the first year of life. At birth and 12 months of age, the questionnaires were completed by mothers with the assistance of the midwives in the hospitals. When children were at 4 and 8 months of age, the questionnaires sent by mail were completed and returned back by the parents within 1 month after the distribution. If needed, parents could ask for assistance to complete the questionnaire by telephoning the midwives. The response rate of our study was 90%. At the age of 12 months, the children were offered a clinical examination.

### Assessment of exposures to air pollutants

Table 1 describes *in utero* and first year of life exposures to air pollutants assessed by standardised questionnaires and their temporal sequence with asthma phenotypes, namely, prenatal maternal smoking and *in utero* exposure to traffic-related air pollution and exposures to heavy parental smoking, traffic-related air pollution, dampness, visible moulds, bleaching agents, house dust mites, pets and domestic heating in the first year of life.

**Table 1:** ***In utero *and first year of life exposures to air pollutants sources and their temporal sequence with asthma phenotypes in children of the EDEN mother-child cohort study**

**Period of life/Air pollutant and related sources**	**Corresponding questions**	**Temporal sequence of asthma phenotypes in relation to exposure**	
		**Exposure assessed in the first of life (at the period of the outcome)**	***In utero *exposure (preceding the outcome)**
***In utero *life**			
Prenatal maternal smoking	1. Did you smoke during the first trimester of pregnancy?	No	Yes
	2. Did you smoke during the second trimester of pregnancy?		
	3. Did you smoke during the third trimester of pregnancy?		
Traffic-related air pollution	1. Does your house located near a bus stop or a passageway of trucks?	No	Yes
	2. Does the cars often or continuously pass by your house?		
**First year of life**			
Heavy parental smoking in the last 12 months	1. Did you smoke at present?	Yes	No
	2. An answer of “more than 20” to the question “How many cigarettes do you smoke every day?”		
Traffic-related air pollution in the last 12 months	1. Do you live less than 200 meters away from a road with heavy traffic?	Yes	No
	2. Notably, Google Earth was used to check the validity of maternal reporting by taking into account a 200 m circular buffer and more than 10,000 vehicles per day (traffic volumes were provided by local authorities) using the home address as reference..		
Dampness in the last 12 months	Are there any problems of dampness, moisture or condensation that have often, or permanently, existed in the house since the birth of the child?	Yes	No
Visible moulds in the past 12 months	Are there any visible moulds that have often, or permanently, existed in the house of the child since the birth of the child?	Yes	No
Bleaching agents in the past 12 months	Do you often clean with or use the bleaching agents in the presence of the child?	Yes	No
House dust mites in the past 12 months	Has your child frequently been in contact with carpets, or glazing, or curtains?	Yes	No
Contact with cats in the past 12 months	1. Was there a new cat in your house and (or) did you let the cat enter the sleeping room of the child?	Yes	No
	2. Did your child contact with cats outside your house at least once per week?		
Contact with dogs in the past 12 months	1. Was there a new dog in your house and (or) did you let the dog enter the sleeping room of the child?	Yes	No
	2. Did your child contact with dogs outside your house at least once per week?		
Type of domestic heating in the past 12 months	Which is your main type of heating in the house: gas, petroleum, wood or others?	Yes	No

### Assessment of asthma phenotypes

An enriched version of the International Study of Asthma and Allergies in Childhood (ISAAC) questionnaire was used to define asthma phenotypes. In order to cover the spectrum of asthma outcomes fully in the first year of life, both single and multi-dimensional asthma phenotypes (as defined in Table 2) were assessed.

**Table 2:** Asthma phenotypes in the first year of life in the children of the EDEN mother-child cohort study

**Asthma phenotypes**	**Corresponding questions**	**Periods**
**Single phenotypes**		
Ever bronchiolitis	Question 1: Did your child suffer from bronchiolitis during the last 4 months?	0-4 months, 4–8 months and 8–12 months
Ever wheezing	Question 2: Did your child ever have wheezing or whistling in the chest after the birth?	0-8 months, 8–12 months
	Question 3: Did your child ever have wheezing or whistling in the chest during the last 4 months?	
Ever doctor-diagnosed asthma	Question 4: Did your child have a diagnosed asthma during the last 4 months?	0-4 months, 4–8 months and 8–12 months
**Multi-dimensional phenotypes**		
Bronchiolitis and wheezing	Questions 1, 2, 3	0-8 months, 8–12 months
Doctor-diagnosed asthma with a history of bronchiolitis	Questions 1, 4	0-4 months, 4–8 months and 8–12 months
Doctor-diagnosed asthma with wheezing	Questions 2, 3, 4	0-8 months, 8–12 months
Doctor-diagnosed asthma with wheezing and a history of bronchiolitis	Questions 1, 2, 3, 4	0-8 months, 8–12 months

### Statistical analysis

This included 3 steps: 1) classical descriptive analysis; 2) analytical analysis for the estimation of the adjusted odds ratios between exposures and asthma phenotypes; 3) the estimation of the adjusted population attributable risk for exposures having statistically significant relationships with the prevalences of asthma phenotypes.

#### Descriptive analysis

The population characteristics were presented by means and percentages. Chi-square test was used to analyze differences of proportion of exposures and asthma phenotypes between boys and girls. Morbidity associated with asthma phenotypes was defined in terms of prevalence (number of cases/number of children at risk at the considered period) and incidence (number of new cases/number of children at risk at the considered period after having excluded those that developed the same asthma phenotype in the previous periods).

#### Analytical analysis

The investigation of the relationships between exposures and asthma phenotypes was conducted using two different models according to whether exposure was assessed simultaneously with health outcome or whether it preceded the health outcome.

1) Exposure assessed simultaneously with asthma

Multivariable logistic regression was implemented to estimate odds ratios (OR) of the relationships between all exposure variables together and the prevalence of each asthma phenotype during the first year of life after adjusting for confounders. Potential confounders included study centre, maternal occupation, maternal age at recruitment, maternal pre-pregnancy body mass index (BMI) as estimated by weight in kilograms divided by the square of the height (kg/m^2^), child’s birth weight, cesarean delivery, preterm birth, breastfeeding, siblings, gender and family history of asthma, eczema, allergic rhinitis or food allergy.

2) Exposure assessed before asthma development

The marginal model [25] was used to analyze the associations of prenatal maternal smoking and *in utero* exposure to traffic-related air pollution together with the prevalence of each asthma phenotype at 3 survey time points (at 4, 8 and 12 months of age) after adjustment for confounders. The parameters of the marginal models were estimated by the Generalized Estimating Equation (GEE) approach, using exchangeable working correlation matrices. In order to study the relationships between the exposures, i.e., prenatal maternal smoking and *in utero* exposure to traffic-related air pollution, and each health outcome in the GEE model, we introduced a variable of time and tested the statistical significance of the presence of interactions between this time variable (=1 if at the age of 4 months, =2 if at the age of 8 months, =3 if at the age of 12 months) and each exposure, adjusting for confounders. As the interaction terms were not significant (*P*-value > 0.05) in the models, they were consequently dropped. In the next step, we studied the associations of all exposures together with each health outcome, including the time variable and confounders. This enabled to estimate the impact of time on the associations between the exposures and each health outcome.

#### Adjusted population attributable risk

The impact of each exposure on asthma phenotypes was estimated by the adjusted Population Attributable Risk (aPAR), the fraction of the total disease experienced in the population that would not have occurred if the effect associated with the risk factor of interest was absent [26]. The aPARs were calculated with the formula provided by Bruzzi [26]:

$$\text{aPAR}=1-\sum \left(\rho_{j}/RR_{j}\right)$$

ρ_*j*_ is the proportion of an asthma phenotype within the stratum *j*. The multivariable logistic regression or GEE model-based odds ratios were used to estimate the relative risk for stratum *j* compared with the baseline stratum (*RR*_*j*_). When the model-based odds ratio was less than 1.0, the aPAR estimate was negative, representing a potentially protective effect. The lower and upper 95% confidence interval (95% CI) of odds ratios for each stratum was used to estimate the lower and upper bounds of aPAR.

Confounders had been identified when the addition of a covariate to the model changed the odds ratio (OR) by 10%, without considering statistical significance. Effect modifiers had been identified when a covariate had a statistically significant interaction (*P*-value < 0.05) with the exposure as tested with the logistic model or the statistic of Wald chi-square in the case of the GEE model.

All statistical tests were performed using SAS® statistical software version 9.2 (SAS® Institute Inc, Cary, North Carolina). All *P*-values were two-tailed. *P* < 0.05 was considered statistically significant.

## Results

### Characteristics of study population

The characteristics of the study population and the potential confounders are described in Table 3. The number of children was equally distributed between two study centres and according to gender. There were no differences for the characteristics between boys and girls (*P* > 0.05), except for study centre, cesarean delivery and birth weight.

**Table 3:** Characteristics of the study population according to gender in the children of the EDEN mother-child cohort study

**Variables**	**All (N = 1765)**	**Boys (n = 918)**	**Girls (n = 847)**	P^*^
**Study centre, n (%)**				
Poitiers	866(49.07)	490(53.38)	376(44.39)	<0.01
Nancy	899(50.93)	428(46.62)	471(55.61)	
**Family income (€/month), n (%)**				
Low(≤1500)	262(14.84)	140(15.25)	122(14.40)	0.15
Middle(1501–3000)	1001(56.71)	534(58.17)	467(55.14)	
High( >3001)	492(27.88)	237(25.82)	255(30.11)	
**Maternal education attainment, n (%)**				
Less than high school	104(5.89)	55(5.99)	49(5.79)	0.97
High school	683(38.70)	352(38.34)	331(39.08)	
College/University or more	950(53.82)	492(53.59)	458(54.07)	
**Maternal occupation, n (%)**				
Blue-collar (laborers)	81(4.59)	49(5.34)	32(3.78)	0.24
White-collar (occupations of intermediate, administrative, sales and customers and personal services)	1339(75.86)	688(74.95)	651(76.86)	
Professional (professional occupations, managers and senior officials)	217(12.29)	108(11.76)	109(12.87)	
**Maternal age at recruitment (years), (Mean ± SD**^**†**^**)**	30.64 ± 4.81	30.70 ± 4.81	30.58 ± 4.81	0.61
**Maternal pre-pregnancy body mass index, n (%)**				
Underweight (<18.50 kg/m^2^)	136(7.71)	74(8.06)	62(7.32)	0.80
Normal (18.50-24.99 kg/m^2^)	1139(64.53)	597(65.03)	542(63.99)	
Overweight (25.00-29.99 kg/m^2^)	305(17.28)	156(16.99)	149(17.59)	
Obese (≥30.00 kg/m^2^)	149(8.44)	73(7.95)	76(8.97)	
**Cesarean delivery, n (%)**	278(15.75)	162(17.65)	116(13.70)	0.02
**Preterm birth**^‡^**, n (%)**	428(24.25)	231(25.16)	197(23.26)	0.35
**Birth weight (grams), (Mean ± SD)**	3285.56 ± 506.65	3350.52 ± 529.41	3214.89 ± 470.89	<0.01
**Breastfeeding, n (%)**				
Never	795(45.04)	436(47.49)	359(42.38)	0.07
≤ 4 months	550(31.16)	279(30.39)	271(32.00)	
> 4 months	420(23.80)	203(22.11)	217(25.62)	
**Siblings, n (%)**	962(54.50)	505(55.01)	457(53.96)	0.68
**Family history of asthma, eczema, allergic rhinitis or food allergy, n (%)**	912(51.67)	484(52.72)	428(50.53)	0.33

^*^P, the *P*-value for t-test or chi-square test between boys and girls;

^†^ SD, standard deviation;

^‡^Maternal gestational age < 37 weeks.

### Exposures to air pollutants

Table 4 describes the frequency of the exposures to air pollutants in the study population. During *in utero* life, 25% of the children had been exposed to maternal smoking and 28% of their mothers had lived close to a road with heavy traffic. Similar figures were found for exposure to traffic-related air pollution in the first year of life (21%), but not for passive smoking (13% exposed to heavy parental smoking). Exposures did not differ between boys and girls (*P* > 0.05).

**Table 4:** ***In *utero and first year of life exposures to air pollutants sources in the children of the EDEN study population according to gender**

**Variables**	**All (N = 1765)**		**Boys (n = 918)**		**Girls (n = 847)**		P^*^
	**n**	**(%)**	**n**	**(%)**	**n**	**(%)**	
***In utero*life**							
Prenatal maternal smoking	450	25.50	228	24.84	222	26.21	0.48
Traffic-related air pollution	501	28.39	246	26.80	255	30.11	0.10
**First year of life**							
Heavy parental smoking in the last 12 months	227	12.86	113	12.31	114	13.46	0.75
Traffic-related air pollution in the last 12 months	372	21.08	201	21.90	171	20.19	0.61
Dampness in the last 12 months	86	4.87	46	5.01	40	4.72	0.90
Visible moulds in the last 12 months	76	4.31	41	4.47	35	4.13	0.84
Bleaching agents in the last 12 months	102	5.78	50	5.45	52	6.14	0.46
House dust mites in the last 12 months	209	11.84	117	12.75	92	10.86	0.29
Cats in the past 12 months	156	8.84	79	8.61	77	9.09	0.75
Dogs in the past 12 months	117	6.63	65	7.08	52	6.14	0.45
Domestic heating in the last 12 months							
Electricity	353	20.00	187	20.37	166	19.60	0.24
Gas	736	41.70	383	41.72	353	41.68	
Petroleum	314	17.79	160	17.43	154	18.18	
Wood	79	4.48	47	5.12	32	3.78	
Others	45	2.55	30	3.27	15	1.77	

^*^P, the *P*-value for chi-square test between boys and girls.

### Asthma phenotypes in the first year of life

The prevalences and incidences of each asthma phenotype at the 3 survey time points (at ages of 4, 8 and 12 months, respectively) are presented in Table 5. In terms of single asthma phenotypes, ever bronchiolitis was the commonest condition at each survey, followed by ever wheezing and then ever doctor-diagnosed asthma. The most frequent multi-dimensional phenotype was constituted by bronchiolitis with wheezing. Almost 6% of children had suffered from all the 3 single phenotypes (ever bronchiolitis, ever wheezing and ever doctor-diagnosed asthma) in the first year of life.

**Table 5:** Prevalence and incidence of asthma phenotypes in the children of the EDEN study population in the first year of life

**Variables**	**4 months survey**		**8 months survey**		**12 months survey**	
	**Incidence (n,%)**	**Prevalence (n,%)**	**Incidence (n,%)**	**Prevalence (n,%)**	**Incidence (n,%)**	**Prevalence (n,%)**
**Single phenotypes**						
Ever bronchiolitis	197(11.16)	197(11.16)	359(22.90)	556(31.50)	156(12.90)	712(40.34)
Ever wheezing	—	—	—	294(16.66)	91(6.19)	385(21.81)
Ever doctor-diagnosed asthma	17(0.96)	17(0.96)	101(5.72)	118(6.69)	17(1.03)	135(7.65)
**Multi-dimensional phenotypes**						
Bronchiolitis with wheezing	—	—	—	148(8.39)	44(2.49)	301(17.05)
Doctor-diagnosed asthma with a history of bronchiolitis	11(0.62)	11(0.62)	50(2.86)	61(3.46)	4(0.24)	112(6.35)
Doctor-diagnosed asthma with wheezing	—	—	—	92(5.22)	5(0.28)	118(6.69)
Doctor-diagnosed asthma with wheezing and a history of bronchiolitis	—	—	—	48(2.72)	3(0.17)	103(5.84)

### Associations between exposures to air pollutants and asthma phenotypes

#### Exposure assessed simultaneously with asthma

Applying the multivariable logistic regression models yielded that various asthma phenotypes were positively associated with heavy parental smoking, traffic-related air pollution and dampness in the last 12 months and negatively associated with contact with cats and domestic wood heating in the last 12 months (see Additional file 1: Table S1). No statistically significant associations (*P* > 0.05) were found between asthma outcomes and visible moulds, bleaching agents, house dust mites, contact with dogs, domestic gas heating, or domestic petroleum heating in the last 12 months (data not showed). A statistically significant interaction between gender and traffic-related air pollution in the last 12 months was found in the multivariable logistic model with ever wheezing as the dependent variable (*P* < 0.01).

#### Exposure preceding asthma development

The application of the marginal model, taking into account the evolution of asthma phenotypes, indicated that prenatal maternal smoking and *in utero* exposure to traffic-related air pollution were associated with ever bronchiolitis (aOR = 1.39 (95% CI: 1.10-1.76) for prenatal maternal smoking, aOR = 1.51 (95% CI: 1.18-1.92) for *in utero* exposure to traffic-related air pollution), but only a trend (with aOR > 1) was found between *in utero* exposures and other asthma phenotypes (data not shown). There was no statistical significance (*P* > 0.05) for the interaction term between exposure to the risk factors and time in the models. The aOR for time was 2.43 (95% CI: 2.28-2.60) for the model describing the relationships between *in utero* exposures and ever bronchiolitis.

#### Adjusted population attributable risk

aPARs varied from 17.78% for exposure to traffic air pollution in the last 12 months to −14.41% for contact with cats in the last 12 months, both in the case of doctor-diagnosed asthma with a history of bronchiolitis (see Additional file 1: Table S1). When considering *in utero* life, aPAR was 7.60% for prenatal maternal smoking and 10.81% for *in utero* exposure to traffic-related air pollution, both in relation to the asthma phenotype of ever bronchiolitis.

## Discussion

In our study, passive smoking and exposure to traffic-related air pollution, both *in utero* life and in the first year of life, and dampness were found to be risk factors for various asthma phenotypes in the first year of life, while contact with cats and domestic wood heating were found to be protective against asthma in the first year of life. Some studies had observed the effects of air pollutants and related sources that we considered in our study. Results of the SIDIRA-2 study (Italian Studies on Respiratory Disorders in Children and the Environment-2nd phase) had showed that passive smoking, high traffic and dampness were risk factors for asthma in children aged 6–7 years [27]. Our data further support the associations in the first year of life.

Evidence for the association between asthma and prenatal and postnatal parental smoking is fairly consistent [10,28-30]. Recently, a systematic review of prospective studies published between 1997 and February 2011 indicated that both prenatal maternal smoking and postnatal maternal smoking were associated with wheezing in the first 2 years of life (OR = 1.41 (95% CI: 1.19-1.67) and OR = 1.70 (95% CI: 1.24-2.35), respectively) [30]. In our study, prenatal maternal smoking had a greater impact on childhood asthma than postnatal heavy parental smoking. This could be explained by the fact that the proportion of prenatal maternal smoking (25%) is higher than that of heavy parental smoking in the last 12 months (13%) in our population. Notably, results of a recent French national prenatal survey showed that 24% of women smoked during pregnancy [31], which is in agreement with our data. Furthermore, compared to postnatal passive smoking, prenatal maternal smoking is more likely to reduce respiratory function after birth [32], which would consequently have a greater impact on the development of asthma.

The association between asthma and traffic-related air pollution has been well documented in the literature [21,27,33-37], but few studies have investigated the association between traffic-related air pollution and asthma in the first year of life. In a previous case–control study in 5 French metropolitan areas, results indicated that traffic related pollutants might be associated with the incidence of asthma in children aged 0–3 years old [37]. In our study, results showed that there were robust associations between *in utero* and first year of life exposure to traffic-related air pollution and asthma phenotypes in the first year of life. Furthermore, the impact of traffic-related air pollution on asthma phenotypes in the first year of life was greater than that of other risk factors even after adjustment for potential confounders, which is consistent with the results of the SIDIRA-2 study previously described [27]. One asset of our study is that we used Google Earth® and traffic volume provided by local authorities at mothers’ address to check the validity of the subjectively measured exposure to traffic and thus minimise the exposure misclassification. The subjectively measured variable of traffic-related air pollution showed high concordance with the Google Earth®-derived variable, with up to 92% of children were well classified. Only 5% of the children whose mother reported traffic-related air pollution were not exposed according to the Google Earth®-derived variable, whereas 3% of the children that did not declare to be exposed were exposed according to this objectively assessed variable. Some studies have compared the subjectively measured exposure to traffic with objectively assessed exposures, implying that both have their own advantages and no method could be *per se* considered as the gold standard [38]. In the USA, Gauderman *et al.* reported that the simple and widely available indicator of the distance between living house and freeway was as strongly and precisely associated with childhood asthma as were more complex objective estimates based on dispersion models [34].

Interestingly, our data further confirmed the findings from previous studies having related bronchiolitis to passive smoking [39] and air pollution [40,41]. This is important in terms of public health because bronchiolitis is an important phenotype of asthma in early life [42].

More consistently, several investigations showed a positive association between dampness and asthma, although very few have considered it in the first year of life. In the DRIAS study (Respiratory Symptoms in children and the Environment in Sardegna, Italy), results showed that exposure to dampness during the first year of life was associated with increased prevalence of current wheeze for primary school children (OR = 1.96; 95% CI: 1.34-2.88) [14]. Similarly, a more recent review implied that dampness was the risk factor for respiratory and allergic health in birth cohorts [43]. However, because of recall bias in the case of questionnaire investigation, the relationship between dampness in the last 12 months and asthma phenotypes in the first year of life needs to be further confirmed. Contrarily to other investigations [9], we did not find any relationship between exposure to house dust mite and the prevalences of asthma phenotypes, probably because the sources (carpets, or glazing, or curtains) we considered were not sufficient.

Our data showed that contact with cats in the last 12 months may be protective against asthma in the first year of life. This is in agreement with previous studies [17]. A potential mechanism was proposed by Platts-Mills *et al*. who indicated that contact with cats could induce IgG and IgG4 antibody responses, which is protective against the development of asthma [44].

In our study, domestic wood heating in the last 12 months was found to be negatively associated with asthma phenotypes in the first year of life. Among other authors, Kilpeläinen *et al.* investigated the association between early life exposure to wood stove heating and subsequent asthma development, results showed that a farm environment was the main confounding factor for the association between wood stove heating and asthma [45]. However, in the present study, the associations between domestic wood heating and asthma phenotypes were not confounded by the rural environment. This is in agreement with the fact that in France wood begins to be widely used for heating even in the urban and peri-urban settings because of its low cost. In addition, it is publicly supported in terms of tax reductions.

The major limitation of our study is that exposures and health outcomes were assessed by questionnaires, without objective assessments. However, our data collection was prospective and the mothers replied for well-standardised questionnaires that were previously validated. In addition, our findings are consistent with those of other studies conducted in other Western European countries and based on questionnaire investigation [14,36,37]. Another limitation of our study is that the exposure to risk factors was assessed simultaneously with asthma outcomes except for prenatal maternal smoking and *in utero* exposure to traffic-related air pollution. Therefore, the causal link implied by our study should be interpreted with caution. Lastly, the questions for assessing *in utero* and first year of life exposure to traffic-related air pollution were different (as showed in Table 1). This is because, after the survey time point of at birth, in order to take results from recent studies into account [46,47], we modified the questionnaire and assessed distances of the dwellings to major road, and in particular the 200 m circular buffer with which a risk of asthma had been associated. As a consequence, responses for *in utero* and first year of life exposure to traffic were not exactly comparable. In addition, the response rates varied from one survey to the other. Indeed, for some mothers, it was difficult to figure out the exact circular buffer and there were more missing values for the variable of first year of life exposure to traffic-related air pollution than for the variable of *in utero* exposure to traffic-related air pollution. Notably, some mother-child pairs changed their residences after the birth of the child. However, in spite of these differences, the associations between asthma phenotypes and the exposure to traffic-related air pollution *in utero* life and in the first year of life are consistent.

The strength of our investigation lies in the fact that the sample is large and drawn from the general population and that we use standardised instruments for the assessment of both exposure and asthma phenotypes. The fact that our investigation focuses on the first year of life which is crucial for the development of asthma using the data of a birth cohort constitutes an added value. In addition, we used different models to analyse the relationships between asthma outcomes and *in utero* and first year of life exposures respectively, taking into account the temporal sequence in relation to asthma outcomes. We also considered the potential confounders and effect modifiers in the models to show the independent effects of exposures *in utero* life and in the first year of life. Lastly, several asthma phenotypes covering a large spectrum of asthma outcomes in early childhood were included thus reducing misclassification of asthmatic cases.

## Conclusions

In our study, asthma outcomes in the first year of life were found to be positively related to maternal smoking, traffic-related air pollution and dampness, but negatively associated with contact with cats and domestic wood heating. Furthermore, *in utero* exposures to passive smoking and traffic-related air pollution were both related to the development of asthma phenotypes in the first year of life. After adjusting for potential confounders, traffic-related air pollution had a greater impact on the development of asthma phenotypes compared to other factors. Interventional studies need to be implemented to evaluate the consequences of the avoidance or the promotion of these factors.

## Consent

Written informed consent was obtained from all participating women for publication of this report and any accompanying images.

## Abbreviations

OR: Odds ratio; aOR: Adjusted odds ratio; aPAR: Adjusted Population Attributable Risk; BMI: Body mass index; SD: Standard deviation; NO2: Nitrogen dioxide; PM10: Particulate matter with an aerodynamic diameter below 10 μm.

## Competing interests

The authors declare that they have no competing interests.

## Authors’ contributions

IAM conceived the study. NB and IAM participated in the data collection. CZ performed statistical analysis and data interpretation with the assistance of NB and SB. CZ wrote the manuscript with the co-supervision of IAM and TZ. All authors read and approved the final manuscript.

## Pre-publication history

The pre-publication history for this paper can be accessed here:

http://www.biomedcentral.com/1471-2458/13/506/prepub

## Supplementary Material

Additional file 1: Table S1 Association between exposures to air pollutants sources and asthma phenotypes in the first year of life in children of the EDEN mother-child cohort study. Click here for file

## References

[B1] Anthracopoulos MB Antonogeorgos G Liolios E Triga M Panagiotopoulou E Priftis KN Increase in chronic or recurrent rhinitis, rhinoconjunctivitis and eczema among schoolchildren in Greece: three surveys during 1991–2003 Pediatr Allergy Immunol 2009 20 180 186 10.1111/j.1399-3038.2008.00752.x 18433422

[B2] Eder W Ege MJ von Mutius E The asthma epidemic N Engl J Med 2006 355 2226 2235 10.1056/NEJMra054308 17124020

[B3] Russell G The childhood asthma epidemic Thorax 2006 61 276 278 10.1136/thx.2005.052662 16565264 PMC2104623

[B4] March ME Sleiman PM Hakonarson H The genetics of asthma and allergic disorders Discov Med 2011 11 35 45 21276409

[B5] Renz H Conrad M Brand S Teich R Garn H Pfefferle PI Allergic diseases, gene-environment interactions Allergy 2011 66 Suppl 95 10 12 21668842 10.1111/j.1398-9995.2011.02622.x

[B6] Annesi-Maesano I Epidemiology of asthma in the world and in France Rev Prat 2011 61 329 335 21563406

[B7] Sly PD The early origins of asthma: who is really at risk? Curr Opin Allergy Clin Immunol 2011 11 24 28 10.1097/ACI.0b013e328342309d 21150438

[B8] Heinrich J Influence of indoor factors in dwellings on the development of childhood asthma Int J Hyg Environ Health 2011 214 1 25 10.1016/j.ijheh.2010.08.009 20851050

[B9] Gaffin JM Phipatanakul W The role of indoor allergens in the development of asthma Curr Opin Allergy Clin Immunol 2009 9 128 135 10.1097/ACI.0b013e32832678b0 19326507 PMC2674017

[B10] Vork KL Broadwin RL Blaisdell RJ Developing asthma in childhood from exposure to secondhand tobacco smoke: insights from a meta-regression Environ Health Perspect 2007 115 1394 1400 17938726 10.1289/ehp.10155 PMC2022647

[B11] Tsai CH Huang JH Hwang BF Lee YL Household environmental tobacco smoke and risks of asthma, wheeze and bronchitic symptoms among children in Taiwan Respir Res 2010 11 11 10.1186/1465-9921-11-11 20113468 PMC2828425

[B12] Hugg TT Jaakkola MS Ruotsalainen RO Pushkarev VJ Jaakkola JJ Parental smoking ensitiz and effects of tobacco smoke on children’s health in Finland and Russia Eur J Public Health 2008 18 55 62 10.1093/eurpub/ckm053 17569700

[B13] Jaakkola JJ Hwang BF Jaakkola N Home dampness and molds, parental atopy, and asthma in childhood: a six-year population-based cohort study Environ Health Perspect 2005 113 357 361 15743728 10.1289/ehp.7242 PMC1253765

[B14] Pirastu R Bellu C Greco P Pelosi U Pistelli R Accetta G Biggeri A Indoor exposure to environmental tobacco smoke and dampness: respiratory symptoms in Sardinian children—DRIAS study Environ Res 2009 109 59 65 10.1016/j.envres.2008.09.002 18952207

[B15] Larsson M Hägerhed-Engman L Moniruzzaman S Janson S Sundell J Bornehag CG Can we trust cross-sectional studies when studying the risk of moisture-related problems indoor for asthma in children? Int J Environ Health Res 2011 21 237 247 10.1080/09603123.2010.533368 21745019

[B16] Pohlabeln H Jacobs S Böhmann J Exposure to pets and the risk of allergic symptoms during the first 2 years of life J Investig Allergol Clin Immunol 2007 17 302 308 17982922

[B17] Dharmage SC Lodge CL Matheson MC Campbell B Lowe AJ Exposure to cats:update on risks for sensitization and allergic diseases Curr Allergy Asthma Rep 2012 12 413 423 10.1007/s11882-012-0288-x 22878928

[B18] von Mutius E Illi S Nicolai T Martinez FD Relation of indoor heating with asthma, allergic sensitisation, and bronchial responsiveness: survey of children in south Bavaria BMJ 1996 312 1448 1450 10.1136/bmj.312.7044.1448 8664621 PMC2351190

[B19] Binková B Bobak M Chatterjee A Chauhan A Dejmek J Dockery DW Everard M Forastiere F Gilliland F Holgate S Johnston S Maynard R Raaschou-Nielsen O Samet J Skerrett PJ Šram RJ Walters D Weiland S Winneke G Krzyzanowski M Kuna-Dibbert B Effects of air pollution on children’s health and development: a review of the evidence 2005 Geneva: World Health Organization

[B20] Tzivian L Outdoor air pollution and asthma in children J Asthma 2011 48 470 481 10.3109/02770903.2011.570407 21486196

[B21] Gehring U Wijga AH Brauer M Fischer P de Jongste JC Kerkhof M Oldenwening M Smit HA Brunekreef B Traffic-related air pollution and the development of asthma and allergies during the first 8 years of life Am J Respir Crit Care Med 2010 181 596 603 10.1164/rccm.200906-0858OC 19965811

[B22] Morgenstern V Zutavern A Cyrys J Brockow I Koletzko S Krämer U Behrendt H Herbarth O von Berg A Bauer CP Wichmann HE Heinrich J GINI Study Group; LISA Study Group Atopic diseases, allergic sensitization, and exposure to traffic-related air pollution in children Am J Respir Crit Care Med 2008 177 1331 1337 10.1164/rccm.200701-036OC 18337595

[B23] Hulin M Simoni M Viegi G Annesi-Maesano I Respiratory health and indoor air pollutants based on quantitative exposure assessments Eur Respir J 2012 40 1033 1045 10.1183/09031936.00159011 22790916

[B24] Drouillet P Kaminski M De Lauzon-Guillain B Forhan A Ducimetière P Schweitzer M Magnin G Goua V Thiébaugeorges O Charles MA Association between maternal seafood consumption before pregnancy and fetal growth: evidence for an association in overweight women. The EDEN mother-child cohort Paediatr Perinat Epidemiol 2009 23 76 86 10.1111/j.1365-3016.2008.00982.x 19228317 PMC2813432

[B25] Omar RZ Wright EM Turner RM Thompson SG Analysing repeated measurements data: a practical comparison of methods Stat Med 1999 18 1587 1603 10.1002/(SICI)1097-0258(19990715)18:13<1587::AID-SIM141>3.0.CO;2-Z 10407231

[B26] Bruzzi P Green SB Byar DP Brinton LA Schairer C Estimating the population attributable risk for multiple risk factors using case–control data Am J Epidemiol 1985 122 904 914 4050778 10.1093/oxfordjournals.aje.a114174

[B27] Forastiere F Galassi C Biggeri A Richiardi L Baussano I Simoni M Viegi G Gruppo Collaborativo SIDRIA-2 The proportion of respiratory disorders in childhood attributable to preventable and not preventable risk factors Epidemiol Prev 2005 29 Suppl 2 67 69 16128558

[B28] Wu H Romieu I Sienra-Monge JJ del Rio-Navarro BE Anderson DM Dunn EW Steiner LL Lara-Sanchez Idel C London SJ Parental smoking modifies the relation between genetic variation in tumor necrosis factor-alpha (TNF) and childhood asthma Environ Health Perspect 2007 115 616 622 10.1289/ehp.9740 17450233 PMC1852663

[B29] Blizzard L Ponsonby AL Dwyer T Venn A Cochrane JA Parental smoking and infant respiratory infection: how important is not smoking in the same room with the baby? Am J Public Health 2003 93 482 488 10.2105/AJPH.93.3.482 12604500 PMC1447768

[B30] Burke H Leonardi-Bee J Hashim A Pine-Abata H Chen Y Cook DG Britton JR McKeever TM Prenatal and passive smoke exposure and incidence of asthma and wheeze: systematic review and meta-analysis Pediatrics 2012 129 735 744 10.1542/peds.2011-2196 22430451

[B31] Blondel B Kermarrec M Perinatal Situation in France in 2010: First Results of the National Perinatal Survey 2010 Paris: DREES 2011

[B32] Stick SM Burton PR Gurrin L Sly PD LeSouëf PN Effects of maternal smoking during pregnancy and a family history of asthma on respiratory function in newborn infants Lancet 1996 348 1060 1064 10.1016/S0140-6736(96)04446-7 8874457

[B33] Annesi-Maesano I Moreau D Caillaud D Lavaud F Le Moullec Y Taytard A Pauli G Charpin D Residential proximity fine particles related to allergic ensitization and asthma in primary school children Respir Med 2007 101 1721 1729 10.1016/j.rmed.2007.02.022 17442561

[B34] Gauderman WJ Avol E Lurmann F Kuenzli N Gilliland F Peters J McConnell R Childhood asthma and exposure to traffic and nitrogen dioxide Epidemiology 2005 16 737 743 10.1097/01.ede.0000181308.51440.75 16222162

[B35] Nordling E Berglind N Melén E Emenius G Hallberg J Nyberg F Pershagen G Svartengren M Wickman M Bellander T Traffic-related air pollution and childhood respiratory symptoms, function and allergies Epidemiology 2008 19 401 408 10.1097/EDE.0b013e31816a1ce3 18379426

[B36] Pénard-Morand C Raherison C Charpin D Kopferschmitt C Lavaud F Caillaud D Annesi-Maesano I Long-term exposure to close-proximity air pollution and asthma and allergies in urban children Eur Respir J 2010 36 33 40 10.1183/09031936.00116109 20075054

[B37] Zmirou D Gauvin S Pin I Momas I Sahraoui F Just J Le Moullec Y Brémont F Cassadou S Reungoat P Albertini M Lauvergne N Chiron M Labbé A Vesta Investigators Traffic related air pollution and incidence of childhood asthma: results of the Vesta case–control study J Epidemiol Community Health 2004 58 18 23 10.1136/jech.58.1.18 14684722 PMC1757023

[B38] Forastiere F Galassi C Self report and GIS based modelling as indicators of air pollution exposure: is there a gold standard? Occup Environ Med 2005 62 508 509 10.1136/oem.2005.020560 16046601 PMC1741067

[B39] Jones LL Hashim A McKeever T Cook DG Britton J Leonardi-Bee J Parental and household smoking and the increased risk of bronchitis, bronchiolitis and other lower respiratory infections in infancy: systematic review and meta-analysis Respir Res 2011 12 5 10.1186/1465-9921-12-5 21219618 PMC3022703

[B40] Karr CJ Demers PA Koehoorn MW Lencar CC Tamburic L Brauer M Influence of ambient air pollutant sources on clinical encounters for infant bronchiolitis Am J Respir Crit Care Med 2009 180 995 1001 10.1164/rccm.200901-0117OC 19713450

[B41] Clark NA Demers PA Karr CJ Koehoorn M Lencar C Tamburic L Brauer M Effect of early life exposure to air pollution on development of childhood asthma Environ Health Perspect 2010 118 284 290 20123607 10.1289/ehp.0900916 PMC2831931

[B42] Smyth RL Openshaw PJ Bronchiolitis Lancet 2006 368 312 322 10.1016/S0140-6736(06)69077-6 16860701

[B43] Mendell MJ Mirer AG Cheung K Tong M Douwes J Respiratory and allergic health effects of dampness, mold, and dampness-related agents: a review of the epidemiologic evidence Environ Health Perspect 2011 119 748 756 10.1289/ehp.1002410 21269928 PMC3114807

[B44] Platts-Mills T Vaughan J Squillace S Woodfolk J Sporik R Sensitisation, asthma, and a modified Th2 response in children exposed to cat allergen: a population-based cross-sectional study Lancet 2001 357 752 756 10.1016/S0140-6736(00)04168-4 11253969

[B45] Kilpeläinen M Koskenvuo M Helenius H Terho E Wood stove heating, asthma and allergies Respir Med 2001 95 911 916 10.1053/rmed.2001.1175 11716206

[B46] Gilbert NL Woodhouse S Stieb DM Brook JR Ambient nitrogen dioxide and distance from a major highway Sci Total Environ 2003 312 43 46 10.1016/S0048-9697(03)00228-6 12873397

[B47] Cesaroni G Badaloni C Porta D Forastiere F Perucci CA Comparison between various indices of exposure to traffic-related air pollution and their impact on respiratory health in adults Occup Environ Med 2008 65 683 690 10.1136/oem.2007.037846 18203803 PMC2771851

