# Supplementary material for: Modifiable exposures to air pollutants related to asthma phenotypes in the first year of life in children of the EDEN mother-child cohort study
Source: BMC Public Health. 2013 May 24;13:506. doi: 10.1186/1471-2458-13-506 (PMC3671198; doi:10.1186/1471-2458-13-506)

**Additional file 1** – Association between exposures to air pollutants sources and asthma phenotypes in the first year of life in children of the EDEN study

|  |  | **aOR^†^(95% CI)** |  | **aPAR(%)^‡^ (95%CI)** |
| --- | --- | --- | --- | --- |
| **Single phenotypes** |  |  |  |  |
| **Ever bronchiolitis** |  |  |  |  |
| Heavy parental smoking in the last 12 months |  | 1.65(1.16,2.33) |  | 6.50(2.29,9.48) |
| Traffic-related air pollution in the last 12 months |  | 1.18(0.90,1.55) |  | — |
| Dampness in the past 12 months |  | 1.32(0.80,2.18) |  | — |
| Contact with cats in the past 12 months |  | 0.69(0.47,1.03) |  | — |
| Domestic wood heating in the past 12 months |  | 0.63(0.38,1.06) |  | — |
| **Ever wheezing** |  |  |  |  |
| Heavy parental smoking in the past 12 months |  | 1.53(1.06,2.19) |  | 6.56(1.13,10.35) |
| Traffic-related air pollution in the past 12 months**^§^** |  | 1.47(1.09,1.97) |  | 9.67(2.50,14.89) |
| Boys |  | 2.01(1.16,3.48) |  | 13.05(3.58,18.51) |
| Girls |  | 0.56(0.28,1.15) |  | — |
| Dampness in the past 12 months |  | 2.12(1.30,3.46) |  | 4.84(2.13,6.50) |
| Contact with cats in the past 12 months |  | 0.94(0.61,1.46) |  | — |
| Domestic wood heating in the past 12 months |  | 0.53(0.27,1.03) |  | — |
| **Ever doctor-diagnosed asthma** |  |  |  |  |
| Heavy parental smoking in the last 12 months |  | 1.25(0.68,2.29) |  | — |
| Traffic-related air pollution in the last 12 months |  | 1.71(1.08,2.72) |  | 13.52(2.38,20.53) |
| Dampness in the past 12 months |  | 2.19(1.06,4.53) |  | 5.26(0.56,7.54) |
| Contact with cats in the past 12 months |  | 0.27(0.08,0.86) |  | -11.50(-46.65,-0.67) |
| Domestic wood heating in the past 12 months |  | 0.97(0.37,2.50) |  | — |
| **Multi-dimensional phenotypes** |  |  |  |  |
| **Bronchiolitis with wheezing** |  |  |  |  |
| Heavy parental smoking in the last 12 months |  | 1.48(0.99,2.21) |  | — |
| Traffic-related air pollution in the last 12 months |  | 1.44(1.05,1.99) |  | 9.39(1.32,15.25) |
| Dampness in the past 12 months |  | 1.88(1.09,3.24) |  | 4.00(0.72,5.90) |
| Contact with cats in the past 12 months |  | 0.80(0.48,1.33) |  | — |
| Domestic wood heating in the past 12 months |  | 0.31(0.12,0.79) |  | -4.70(-15.14,-0.57) |
| **Doctor-diagnosed asthma with a history of bronchiolitis** |  |  |  |  |
| Heavy parental smoking in the last 12 months |  | 1.24(0.63,2.43) |  | — |
| Traffic-related air pollution in the last 12 months |  | 2.01(1.23,3.30) |  | 17.78(6.54,24.63) |
| Dampness in the past 12 months |  | 1.77(0.76,4.09) |  | — |
| Contact with cats in the past 12 months |  | 0.22(0.05,0.91) |  | -14.41(-72.19,-0.39) |
| Domestic wood heating in the past 12 months |  | 0.46(0.11,1.93) |  | — |
| **Doctor-diagnosed asthma with wheezing** |  |  |  |  |
| Heavy parental smoking in the last 12 months |  | 1.40(0.76,2.58) |  | — |
| Traffic-related air pollution in the last 12 months |  | 1.71(1.05,2.77) |  | 13.27(1.59,20.50) |
| Dampness in the past 12 months |  | 2.21(1.04,4.71) |  | 5.52(0.35,7.95) |
| Contact with cats in the past 12 months |  | 0.31(0.09,0.99) |  | -10.55(-45.04,-0.01) |
| Domestic wood heating in the past 12 months |  | 0.85(0.30,2.42) |  | — |
| **Doctor-diagnosed asthma with wheezing and a history of bronchiolitis** |  |  |  |  |
| Heavy parental smoking in the last 12 months |  | 1.31(0.67,2.59) |  | — |
| Traffic-related air pollution in the last 12 months |  | 1.90(1.14,3.15) |  | 15.92(4.11,23.01) |
| Dampness in the past 12 months |  | 1.90(0.82,4.43) |  | — |
| Contact with cats in the past 12 months |  | 0.23(0.06,0.97) |  | -14.16(-78.77,-0.13) |
| Domestic wood heating in the past 12 months |  | 0.23(0.03,1.68) |  | — |

^†^aOR: odds ratio adjusted by potential confounders of study center, maternal occupation, maternal age at recruitment, maternal pre-pregnancy body mass index (BMI), birth weight, cesarean delivery, preterm birth, breastfeeding, siblings, gender and family history of asthma, eczema, allergic rhinitis or food allergy, according to the multivariable logistic regression models.

^‡^aPAR: adjusted population attributable risk based on multivariable logistic regression models;

**^§^**Interaction between gender and traffic-related air pollution in the last 12 months.


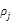

Supplement: Additional file 1: Table S1 — Association between exposures to air pollutants sources and asthma phenotypes in the first year of life in children of the EDEN mother-child cohort study. [file 1471-2458-13-506-S1.doc]
